# Using isotemporal substitution to predict the effects of changing physical behaviour on older adults’ cardio-metabolic profiles

**DOI:** 10.1371/journal.pone.0224223

**Published:** 2019-10-23

**Authors:** Declan J. Ryan, Jorgen Antonin Wullems, Georgina Kate Stebbings, Christopher Ian Morse, Claire Elizabeth Stewart, Gladys Leopoldine Onambele-Pearson

**Affiliations:** 1 Musculoskeletal Sciences & Sport Medicine (MSSM) Research Centre, Department of Sport & Exercise Science, Manchester Metropolitan University, Manchester, United Kingdom; 2 Science, University of Northampton, Northampton, Northamptonshire, United Kingdom; 3 Musculoskeletal Rehabilitation Research Group, Department of Rehabilitation Sciences, KU Leuven, Leuven, Flanders, Belgium; 4 Research Institute for Sport and Exercise Sciences, Liverpool John Moores University, Liverpool, Merseyside, United Kingdom; University of Idaho, UNITED STATES

## Abstract

**Background:**

It has been advocated that older adults should concomitantly spend less time in sedentary behaviour (SB), and engage in sufficient physical activity (PA), to reduce their risk of cardio-metabolic diseases. However, it is not clear what intensity of PA must be done to offset SB engagement.

**Aim:**

Model how cardio-metabolic profiles could change if older adults replaced an hour per day (hr·day^-1^) of a physical behaviour intensity with 1 hr·day^-1^ of another physical behaviour of a different intensity.

**Methods:**

Older adults (*n* = 93, 60–89 years old, 55% female) wore a thigh-mounted triaxial accelerometer for seven consecutive free-living days to estimate mean daily hourly engagement in SB, Standing, Light Intensity PA (LIPA), sporadic moderate to vigorous physical activity (sMVPA, bouts <10 continuous minutes), and 10-minute MVPA (_10_MVPA, bouts ≥10 continuous minutes. Fasting whole blood concentration of plasma glucose, triglyceride, total cholesterol, and glycated haemoglobin (%), along with serum concentration of lipoprotein lipase (LPL), interleukin-6 (IL-6), and procollagen III N-terminal propeptide (PIIINP) were measured.

**Results:**

Isotemporal Substitution, with covariate adjustment, suggested that: total cholesterol concentration could theoretically decrease when 1 hr·day^-1^ of SB is replaced with Standing, when 1 hr.day^-1^ of LIPA is replaced with Standing, and when 1 hr·day^-1^ of sMVPA is replaced with Standing. Triglyceride concentration theoretically decreased when 1 hr·day^-1^ of SB, Standing, LIPA, or sMVPA is replaced with _10_MVPA. Triglyceride concentration theoretically increases when 1 hr·day^-1^ of _10_MVPA is replaced with SB, Standing, or LIPA. No associations with time reallocation appears to exist for LPL, HbA1c, IL-6, and PIIINP.

**Conclusion:**

The type of physical behaviour being replaced could be crucial for total cholesterol maintenance. Engagement in _10_MVPA could be necessary to improve triglyceride concentration.

## Introduction

In the past, attitudes to physical activity recommendation for older adults was simply that “some physical activity is better than none” [1]. More recently, guidelines have been more detailed so that in the United Kingdom for instance, the recommendations are that older adults should engage in at least 150 minutes per week of moderate to vigorous physical activity (MVPA) through the accumulation of bouts of at least 10 minutes (_10_MVPA) [1]. Reaching this target is thought to reduce the risk of cardiovascular mortality by 35% [2]. However based on objective monitoring of physical behaviour within older adult cohorts, where cardiovascular disease is one of the leading causes of death [3], less than 15% of the population are thought to attain the 150 minutes per week _10_MVPA target [4, 5]. With such low attainment rates for the _10_MVPA recommendation within older adult populations, it is pertinent to find and advocate, alternative physical behaviour interventions that could improve cardiovascular health for adults who do not or cannot attain the _10_MVPA recommendation.

Epidemiological studies have suggested that physical behaviour at intensities below MVPA could modulate cardiovascular disease risk factors, such as plasma triglycerides and glucose [6–8]. However, implementation of these findings by end-users would require a change in their habitual physical behaviour profile that, in a 24-hour day, would reduce the amount of time they could spend engaging in other physical behaviours. Ultimately, physical behaviour change is not just about the newly engaged physical behaviour but also about the physical behaviour that is being replaced, and the collective effects that the changes in these two physical behaviour intensities have on health status. Until recently, physical behaviour epidemiology had failed to account for the time limiting impact of a 24-hour day when predicting how a change in a physical behaviour may affect health status.

Isotemporal Substitution Modelling is a relatively new form of statistical analysis within PB research, first used to predict weight change in middle-aged women (25–42 years) [9]. Since this seminal research, 80 publications have utilised ISM (PubMed, retrieved 26/02/2019). However, there is an apparent lack of studies that examine the associations between time reallocation and cardio-metabolic profile in older adult populations [10].

The adoption of isotemporal substitution modelling (ISM) [9] by physical behaviour researchers has now made it possible to determine how health status may be affected by replacing the time spent engaging in one physical behaviour intensity with another physical behaviour intensity. The growing evidence base of ISM has highlighted that as little as a 10-minute change in physical behaviour profile can affect cardio-metabolic status [11–20]. However, previous ISM studies have ‘objectively’ estimated the engagement in the physical behaviour spectrum with hip, waist, or wrist mounted accelerometers, which are arguably less accurate in terms of classifying posture and thus, cannot reliably distinguish between sedentary behaviour (SB) and standing, for instance, which have shown to have opposing effects on health status [12, 21–23].

Thus, the aim of the current cross-sectional study was to model how older adults’ cardio-metabolic endocrine profiles would change if they replaced an hour per day (1 hr·day^-1^) of a physical behaviour intensity with 1 hr·day^-1^ of another physical behaviour of a different intensity (e.g. replace SB with light intensity physical activity [LIPA]). It was hypothesised that replacing 1 hr·day^-1^ of physical behaviour for one of a higher intensity (in terms of metabolic demands) would improve cardio-metabolic status and *vice versa*.

## Materials and methods

Ethical approval was granted by the Manchester Metropolitan University Exercise and Sports Science Ethics Sub-Committee (Approval Code: 03.11.14(i)). Written informed consent was provided by all participants prior to data collection. Participants were recruited from older adult community groups (e.g. Bridge clubs and Bowls clubs) within a rural, affluent, and highly educated county in England [24, 25] Older adults (≥ 60 years old) were eligible for the study if they were independently mobile (did not require walking aids), were not diabetic, had no current/past history of neurological disorders likely to limit mental/physical function at the time of the study, were not suffering from an un-treated cardiovascular disease. Ninety-three older adults (73.8 ± 6.23 years, 60–89 years, 55% female) were recruited between January 2015 and June 2016. Participants visited the laboratory on two occasions, separated by at least seven days.

### First laboratory visit

During the first laboratory visit, participants were familiarised with the testing protocol that they would undergo during the second laboratory visit. Demographic information, such as height, weight, and current medical prescriptions were also collected. Medication use was presented in the following formats: the number of different medications prescribed for daily use, which could directly influence cardiovascular profile (Directly CVD Meds [n·day^-1^]), the number of different medications prescribed for daily use, which could (in)directly influence cardiovascular profile ((In)directly CVD Meds [n·day^-1^]), the sum daily dosage of inflammatory, (in)directly influencing cardiovascular profile medication (CVD Meds [mg·day^-1^]), daily dose of blood pressure medication (BP Meds [mg·day^-1^]), daily dose of lipid-lowering medication (Lipid-Lowering Meds [mg·day^-1^]).

Participants were fitted with a thigh-mounted triaxial accelerometer (GENEActiv Original, Activinsights Ltd, Kimbolton, UK) on the dominant leg (anterior aspect, 50% of greater trochanter to femoral condyle distance) using two waterproof adhesive patches (3M Tegaderm Film, North Ryde, Australia) to be worn for seven consecutive free-living days. The full details of the accelerometer programming can be found in [26]. Briefly, an in-house developed data analysis software (the Cheshire Algorithm for Sedentarism [CAS]) [27] utilised 60 Hertz sampling frequency, 10 second epoch accelerometer data to determine participant time spent in SB (seated or reclined posture, ≤1.5 Metabolic Equivalent Tasks [METs]), Standing (upright posture, ≤1.5 METs), LIPA (upright posture 1.5–3.0 METs), Sporadic MVPA (sMVPA, upright posture, ≥3.0 METS, for <10 consecutive minutes), and _10_MVPA (upright posture, ≥3.0 METS, for ≥10 consecutive minutes. Nighttime sleeping hours (i.e. daytime naps not included) was self-reported using a written sleep-diary and included within CAS analysis. Where sleeping hours was not reported, raw accelerometer data was cross-referenced to identify prolonged nocturnal reclined posture. Thus, an estimate of when a participant went to bed and woke up on the monitored week was always possible. Three participants were removed from analysis for having <6 days of accelerometer data.

### Second laboratory visit

Participants arrived at the laboratory in an overnight (>10 hours) fasted, hydrated state. Participants refrained from taking medication on the morning of the laboratory visit until the completion of a 10 mL venous blood sample donation. Hydration, medication intake and a light breakfast were then allowed immediately after said sampling.

### Whole blood cardio-metabolic analysis

Immediately following blood donation (i.e. within 3 mins), whole blood analyses of total cholesterol, triglyceride, glucose, and glycated haemoglobin (HbA1c, sub-sample of *n* = 33) using three Accutrend Plus monitor devices and test strips (Roche Diagnostics Limited, Welwyn Garden City, UK) and one HemoCue 501 device and test cartridge (HemoCue, Ängelholm, Sweden), respectively, were carried out. Analyses were performed in triplets and averaged for total cholesterol, triglyceride, and glucose whereas only a single sample was analysed for HbA1c. Accutrend monitors have shown no difference in comparison to clinical laboratory methods in the measurement of total cholesterol, triglyceride, and glucose concentration [28]. Furthermore, the HemoCue 501 has shown good reliability (Coefficient of Variation [CV] <5/0%) and validity (Bland-Altman: 4.4 [95%CI -7.3, 16.2] mmol∙mol^-1^) compared to high performance liquid chromatography ion exchange [29]

The remainder of the whole blood sample was allowed to clot whilst placed on crushed ice in a 10 mL Serum Vacutainer (Becton Dickinson, New Jersey, USA) for less than two hours before centrifugation at 1687 G for five minutes (Z380, Hermle, Gosheim, Germany). Serum was extracted into 1.0 mL aliquots (Eppendorf Ltd, Hamburg, Germany) and stored at -20 ºC until further analyses.

### Serum cardio-metabolic analyses

Concentration of serum lipoprotein lipase (LPL), procollagen III N-terminal propeptide (PIIINP), and interleukin-6 (IL-6) were determined using commercially available enzyme-linked immunosorbent (ELISA) assay kits (LPL: Cell Biolabs Inc., California, USA, PIIINP: Biomatik, Delaware, USA, IL-6: high-sensitivity, Bio-Techne, Minnesota, USA) with a two-fold sample dilution. Manufacturer intra-assay sample CV was 4% (<13% in-house) for LPL, <10% (6.5–9.6% in-house) for PIIINP, and 7.8% (7.4–9.2% in-house) for IL-6. A 96-well spectrophotometer (EL808, BioTek, Vermont, USA) connected to a computer running Gen5 c 1.11 software (BioTek, Vermont, USA) was used to derive protein concentration data.

### Statistical analyses

SPSS version 22 (IBM, New York, USA) was used for statistical modelling. Physical behaviour parameters, measured in mean hrs∙day^-1^ (SB, Standing, LIPA, sMVPA, and _10_MVPA) and the summation of these parameters (Total PB) were used for ISM. Pearson correlation was used to assess multicollinearity between physical behaviour parameters and total PB; no adjustment to the data was made if multicollinearity was present. The largest case of collinearity was between SB and LIPA (*r*^*2*^ = -0.69), which was below the suggested limits of collinearity (*r*^*2*^>0.9) [30]. To illustrate the effect on cardio-metabolic parameters with the replacement of one hour of a physical behaviour with another, the replaced physical behaviour parameter was removed from the linear regression model (forced entry) (i.e. replace SB model: Intercept + (*ẞ1* х Standing) + (*ẞ2* х LIPA) + (*ẞ3* х sMVPA) + (*ẞ4* х _10_MVPA) + (*ẞ5* х Total PB) + Covariates + Error) [30]. Significant predictors in the model illustrate that the replacement of a physical behaviour with the significant physical behaviour could have a predicted effect on the cardio-metabolic parameter. To standardise reporting of the ISM outcome, the removed physical behaviour is mentioned first. For example, *‘the replacement of one hour of SB with standing reduced total cholesterol’* means that SB engagement has been reduced by one hour and replaced with one hour of standing.

ISM was performed without (Model 1) and with (Model 2) covariate adjustment (Directly CVD Meds [n·day^-1^], [In]directly CVD Meds [n·day^-1^], CVD Meds [mg·day^-1^], BP Meds [mg·day^-1^], Lipid-Lowering Meds [mg·day^-1^]). A covariate was included in the ISM if a forced entry linear regression model had highlighted an association between the covariate and the respective cardio-metabolic parameter in a preliminary analysis of the dataset. These covariates were chosen as they have previously been shown to influence the cardio-metabolic parameters of interest in the current study [31–34]. Cardio-metabolic data were naturally LOG transformed if they violated normal distribution (total cholesterol, triglyceride, LPL, IL-6, and PIIINP). Data are presented as beta coefficient (95% Confidence Interval [CI]) unless stated otherwise. Statistical significance was set at *p*≤0.05.

## Results

To enhance reader experience, this study features augmented reality. To access this feature, please download HP Reveal (Hewlett-Packard, California, USA) from your smart phone app store and create a free account. Search for, then follow DrDeclanRyan, scan your phone camera over the figures within this study to view the augmented reality attachments.

The sample population characteristics are detailed below (Table 1). Over 50% of the sample population had lipid markers that were above international recommended thresholds (triglyceride: 56%, total cholesterol: 66%). Whilst for glucose markers, 35% of the sample population had a higher than recommended glucose concentration but no participants had an above threshold HbA1c percentage. Twenty-one percent of the participants were not prescribed medication, whilst 30% and 60% of the population had been prescribed lipid-lowering and blood pressure medication, respectively. Notably, mean _10_MVPA was below 10 mins·day^-1^ (Table 1), this was because 49 participants failed to register a single bout of 10-continuous minutes of MVPA across seven-days of monitoring.

**Table 1:** Participant demographics for the study sample. Displayed as Mean (Standard Deviation) unless stated otherwise.

Variable	Pooled Population
*n*	93
Female (%)	55
Age (years)	73.8 (6.22)
Height (m)	1.65 (0.08)
Mass (kg)	75.9 (13.1)
BMI (kg·m^2^)	27.9 (4.71)
**Covariates**	
Directly CVD Meds (*n*∙day^-1^)†	1.17 (1.52)
(In)directly CVD Meds (*n*∙day^-1^)‡	1.62 (1.81)
Inn+ (In)directly CVD Meds (mg∙day^-1^)¥	157.86 (486.49)
BP Meds (mg∙day^-1^)	9.81 (50.79)
Lipid-Lowering Meds (mg∙day^-1^)	8.12 (16.58)
**Cardio-metabolic Parameters**	
Triglyceride (mmol∙l^-1^)	1.77 (0.81)_m_
Total Cholesterol (mmol∙l^-1^)	5.44 (1.39)_m_
Glucose (mmol∙l^-1^)	5.72 (1.10)_m_
HbA1c (%)	5.29 (0.31)
LPL (pg∙mL^-1^)	113.02 (147.80)_m_
IL-6 (pg∙mL^-1^)	2.72 (2.77)_m_
PIIINP (pg∙mL^-1^)	229.21 (247.97)_m_
**Physical Behaviour Parameters**	
SB (hrs∙day^-1^)	9.65 (1.33)
Standing (hrs∙day^-1^)	1.09 (0.41)
LIPA (hrs∙day^-1^)	1.97 (0.63)
sMVPA (hrs∙day^-1^)	2.57 (0.64)
_10_MVPA (hrs∙day^-1^)	0.08 (0.20)_m_
Total PB (hrs∙day^-1^)	15.4 (4.77)_m_

† Participants are currently prescribed an amount of medication that reduces the risk or treats CVD (i.e. statins, warfarin)

‡ Participants are currently prescribed a medication that may affect the cardiovascular system either directly or as a side effect

¥ Participants are currently prescribed a medication that may affect the cardiovascular system either directly or as a side effect, including inflammatory medication. _m_ Median (IR).

**Fig 1:**
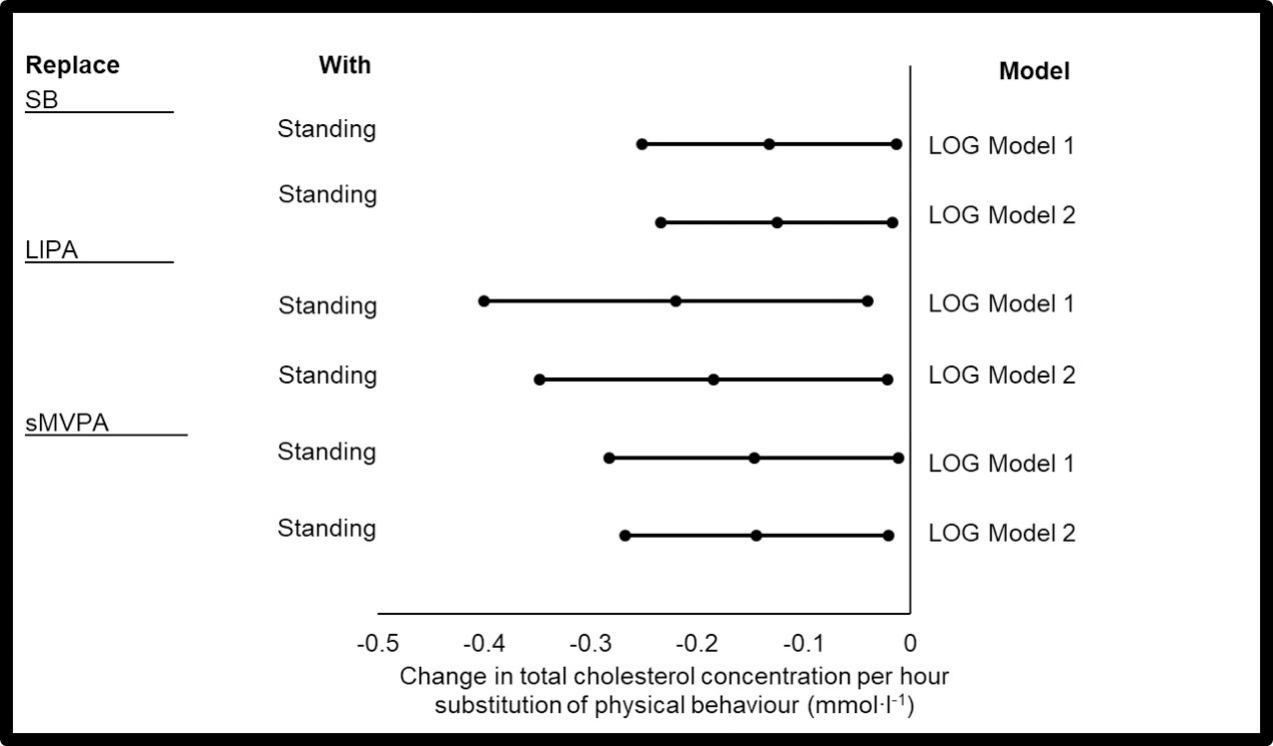
Significant changes in total cholesterol concentration with the replacement of 1 hr·day^-1^ of PB with another. Model 1: no covariate adjustment. Model 2: adjusted for directly CVD medication (n∙day^-1^) and (in)directly CVD medication (n∙day^-1^). Data points represent LOG data (from left to right): -95%CI, beta coefficient, +95%CI. p≤0.05. This figure is Augmented Reality Ready, download HP Reveal from your Smart Phone App Store to access the embedded videos.

**Fig 2:**
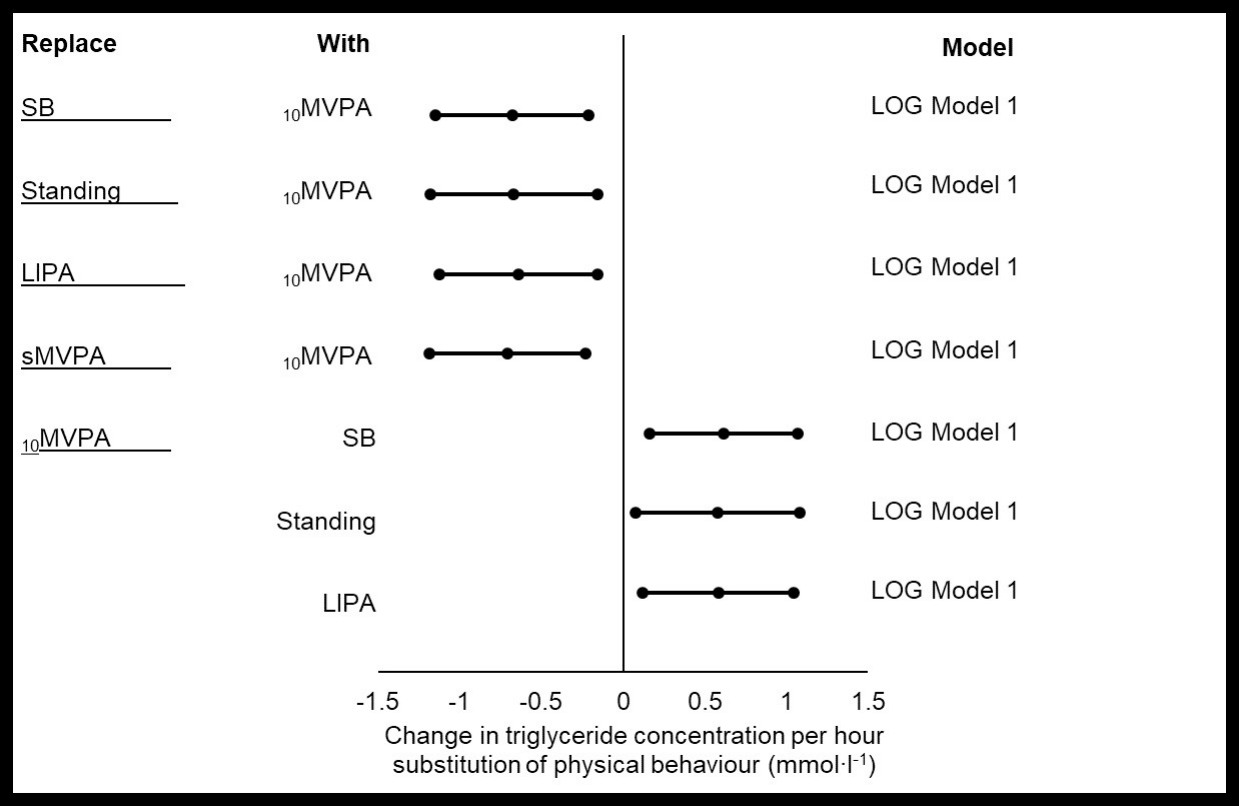
Significant changes in triglyceride concentration with the replacement of 1 hr·day^-1^ of PB with another. Model 1: no covariate adjustment. Data points represent LOG data (from left to right): -95%CI, beta coefficient, +95%CI. p≤0.05. This figure is Augmented Reality Ready, download HP Reveal from your Smart Phone App Store to access the embedded videos.

It is pertinent to note that for simplicity, only significant models are presented in the results section. The complete set of the results is displayed in the Supporting Information (S1–S7 Tables). Significant ISM was present for total cholesterol and triglyceride (Figs 1 and 2) i.e. two out of a possible seven cardio-metabolic markers that were monitored.

### Total cholesterol

Without covariate adjustment, the replacement of 1 hr·day^-1^ of SB with standing was associated with a reduction in total cholesterol by (anti-logged data) 1.14 (95%CI 1.28, 1.01) mmol∙l^-1^ (Fig 1). This is clinically relevant as a 0.5 mmol∙l^-1^ decrease in total cholesterol concentration is associated with a 17% decreased risk in coronary heart disease mortality [35]. The replacement of LIPA with standing was associated with a reduction in total cholesterol (anti-logged data, 1.25 [95%CI 1.49, 1.04] mmol∙l^-1^) (Fig 1). Finally, the replacement of sMVPA with standing was associated with a reduction in total cholesterol by (anti-logged data) 1.15 (95%CI 1.32, 1.01) mmol∙l^-1^ (Fig 1). With adjustment for directly CVD medication (*n*∙day^-1^) and (in)directly CVD medication (*n*∙day^-1^), all significant models (model 1) remained significant (model 2) (Fig 1).

### Triglyceride

There were no covariates for triglyceride. Most notably, the replacement of any PB with _10_MVPA was associated with a decrease in triglyceride concentration by similar amounts, as the change in triglyceride concentration fell between (anti-logged data) 1.89–2.03 (95%CI range: 3.16, 1.17) mmol∙l^-1^ (Fig 2). On the other hand, the replacement of _10_MVPA with any other PB (excluding sMVPA) was associated with an increase in triglyceride concentration by similar amounts (anti-logged data, beta coefficient range: 1.79–1.86 [95%CI range: 1.17, 2.94]) mmol∙l^-1^ (Fig 2). These results are clinically relevant as a 1.0 mmol∙l^-1^ increase in triglyceride concentration is associated with a 14% (95%CI 5, 28%, male) - 37% (95%CI 13, 66%, female) relative risk increase in CVD prevalence [36].

## Discussion

The objective of this study was to mathematically model, using ISM, how a strictly older adult population (60–89 years) could change their cardio-metabolic profile through changes in PB engagement. It was hypothesised that substitution of SB or PA with PA of higher intensity may theoretically improve cardio-metabolic profile whilst the substitution of a higher intensity PA with a lower intensity PA or SB would theoretically worsen cardio-metabolic profile, as has been illustrated previously in young-middle and middle-older adult populations [11, 12, 14]. Indeed the hypotheses were confirmed as the replacement of SB with standing was associated with a decrease in total cholesterol concentration and the replacement of any PB with _10_MVPA was associated with a decrease in triglyceride concentration. Therefore, the results of the current study suggested that using PB interventions could be a useful method for the prevention and treatment of lipid disorders, such as atherosclerosis, which when it manifests in the coronary arteries, is responsible for 44.5% of CVD deaths in UK older adults [3]. However, at face value, some of the total cholesterol results were counterintuitive as the replacement of sMVPA and LIPA with standing was, in fact, associated with a decrease in total cholesterol concentration.

### The two sides to standing

Total cholesterol is comprised of high-density lipid cholesterol (HDL-C), low-density lipid cholesterol (LDL-C), and triglyceride. The theoretical reduction in total cholesterol concentration, after sMVPA and LIPA were replaced with standing in the current model, may be a product of lowered HDL-C, thereby suggesting that standing negatively affects cardio-metabolic status. Indeed, one month of detraining following a LIPA intervention in older adults (mean age 75.5±5.60 years) has been reported to decrease HDL-C concentration whilst LDL-C and triglyceride remained constant [37].

Not only did the modelled replacement of sMVPA and LIPA with standing theoretically reduce total cholesterol within the current study but a decrease in total cholesterol concentration was also associated with SB being replaced with standing. It could be postulated that standing is positively affecting lipoprotein profile in this case, possibly by reducing non HDL-C lipoproteins. This postulation is in agreement with a previous four-day intervention study in young adults (21±2 years), which found non-HDL-C concentration decreased from 2.94±0.47 to 2.65±0.48 mmol·l^-1^ following the replacement of six hours of SB with two hours of standing and four hours of walking, compared to a 14 hrs·day^-1^ of SB day [23]. Meanwhile, there was also a non-significant trend for total cholesterol to decrease from 4.20±0.67 to 3.96±0.50 mmol·l^-1^ (*p* = 0.17) [23]. Although ISM can suggest an intervention, it cannot state how long an intervention can take to have a significant effect on health status. However, along with the results of Duvivier, Schaper [23], it could be suggested that the replacement of one hour of SB with standing could reduce total cholesterol if the intervention lasts longer than four days.

Overall, these findings suggested that standing could affect circulating cholesterol, differentially, depending on which PB parameter it is displacing and thus facilitates the prediction of individualised outcomes for PB change interventions.

### Benefits of prolonged moderate activity

Performing longer bouts of MVPA may induce a chronic physiological effect that protects against physical inactivity, as total cholesterol concentration modelling was predicted to decrease when sMVPA, but not _10_MVPA, was replaced with standing within the current study. In terms of the time frame for adaption in cholesterol profile with the introduction of physical inactivity, previous studies have varied considerably depending on what duration of MVPA bouts were being undertaken prior to physical inactivity. For example, the lipoprotein profile of trained endurance rowers took 29 weeks, following the cessation of exercise (performed in bouts of MVPA ≥10 mins), to become significantly different. Specifically, HDL-C decreased from 0.92±0.09 pre detraining to 0.81±0.11 mmol·l^-1^ post detraining (*p*<0.05, a 0.004 mmol·l^-1^ reduction per week) [38]. Whereas, it took only four weeks until HDL-C concentration was significantly reduced (from 1.42±0.05 to 1.34±0.05 mmol·l^-1^, *p*<0.05, a 0.02 mmol·l^-1^ reduction per week) following the cessation of a four-month interval training intervention (performed in bouts of MVPA <10 mins, 4 × 4 mins MVPA, 3 mins rest) [39]. Despite these previous finding being reported in middle-aged adults, there are similarities in cardio-metabolic change with a reduction in MVPA when compared with the current study. It is likely that engagement in prolonged bouts of MVPA (_10_MVPA) would be beneficial in the preservation of long-term cholesterol profile should an older adult be temporarily forced into a physically inactive lifestyle (i.e. prolonged bedrest due to illness or injury).

Furthermore, the associated reduction in fasted triglyceride concentration when any PB, including sMVPA, was replaced with _10_MVPA in the current study, also strengthens the theorem that prolonged MVPA bouts would improve/maintain lipoprotein profile. In addition, the lack of association on fasted triglyceride concentration when SB was replaced for PA suggests that a focus on reducing physical inactivity is more important than SB reduction when attempting to improve triglyceride profile. Whereas the lack of effect on fasted total cholesterol when _10_MVPA is replaced in the model (and improvements when SB is replaced) could suggest that SB reduction as opposed to physical inactivity reduction could be more important for total cholesterol profile improvements, possibly due to the effects on LDL-C and HDL-C pathway alluded to above. However, intervention studies would be required to confirm or deny the hypotheses engendered through the present modelling exercises.

### No effect of physical behaviour on lipoprotein lipase

Pre-heparin serum LPL concentration was measured within the current study and it is thought to represent whole-body LPL production and the systemic potential to hydrolyse triglycerides [40, 41]. Additionally, pre-heparin serum LPL has been shown to be inversely related to the progression of coronary artery disease in young/middle–older aged populations (Hitsumoto, Ohsawa [42]: 22–79 years, Rip, Nierman [43]: 45–79 years), highlighting that pre-heparin serum LPL is anti-atherogenic. Furthermore, exercise has been shown to increase LPL within humans and rats in all age groups [44–46], thus suggesting that PA can protect against atherosclerosis through the mediation of the LPL pathway. Conversely, the results of the current modelling study suggested that the replacement of any PB may not necessarily affect serum LPL concentration. A possible explanation for these results is that pre-heparin serum LPL is representative of whole-body LPL [40, 41], in which, the expression of LPL within certain sites can portray a pro-atherogenic effect (e.g. artery wall) [47]. Therefore, the representation of both pro and anti-atherogenic LPL within pre-heparin serum LPL may cause ‘noise’ within the ISM, thus nullifying any effects on pro or anti-atherogenic LPL that may exist with PB change. Furthermore, this ‘noise’ is likely to be more pronounced in the older adult population, who already have an increased serum LPL concentration compared to middle aged adults [48] and are more likely to show progression of atherosclerosis, which has been positively associated with LPL concentration within the aorta [49].

### Not statistically significant but clinically significant results

Although the confidence interval range of some of the cardio-metabolic markers suggested no association with PB substitution, there were beta co-efficients and confidence intervals that were large enough to suggest some clinical relevance. Previous findings from Emerging Risk Factors Collaboration [50] suggested that a 1 mmol·l^-1^ increase in fasting plasma glucose was associated with a 12% increase in hazard ratio for coronary heart disease incidents. Within the findings of the current study, replacing _10_MVPA with SB had a beta-coefficient of 0.38 mmol·l^-1^ and an upper confidence interval limit of 2.04 mmol·l^-1^ (S4 Table). This may suggest that some older adults will experience adverse effects of replacing _10_MVPA with SB. It could be that the design of this study may not have been sufficient to detect statistically significant associations, but it should not mean that future studies dismiss further research.

### Strengths and limitations

The current study’s strength include the utilisation of a week-long thigh-mounted accelerometer to accurately detect all physical behaviours including sedentary behaviour, every 60 seconds. Another strength was the fact that the accelerometers were thigh mounted which allowed for the investigation of standing; a physical behaviour which has shown mixed results in randomised cross-over trials in terms of its impact on cardio-metabolic profile during prolonged sitting [22, 23, 51, 52]. Furthermore, the physical behaviour data in our study has allowed the investigation of the importance of the 10-minute criterion for MVPA, which is a current discussion point within the UK physical activity guidelines. Finally, the utilisation of isotemporal substitution analysis is essentially mathematical modelling of potentially effective physical behaviour change guidance, ultimately useful to both practitioners and end-users, as such analyses may assist with individualising physical behaviour programmes. It is nevertheless noted that given the cross-sectional study design, the presence of reverse causality bias for instance, cannot be ruled out. In addition to this, as this study was performed on a relatively small sample, some of the analysis may have been under-powered, increasing the chance of a type II error. An unsurmountable study limitation in this cross-sectional study design, was the fact that we could not stop participants taking pre-existing medications and indeed it would have been unrealistic and unrepresentative of an older population to have only recruited older persons on zero phamaceuticals. Whilst we applied statistical adjustment to this limitation by treating medication use as a covariate, the fact that we reduced this data to the categorical level means that the precision of our adjustment was limited. Future research should conduct this modelling with longitudinal data of a larger older adult sample size. We also recommend that future studies be statistically powered to recruit samples large enough to have sufficient numbers of participants using similar drug therapies. In this way, the amount and type of medication can be recorded and thus used at the ratio/interval level, thereby improving on the quality of adjustments.

## Conclusion

The current study added further evidence to support the 10-minute bout recommendation within the MVPA guidelines of PA for older adults, as well as highlighting that quantitative guidelines are required for reducing SB engagement in older adults. Finally, the current study has potentially shown that a PB can have both a positive and negative effect on cardio-metabolic status (though requiring further investigation), dependent on what PB it is replacing. Thus, advocating the need for individualised interventions, where possible, that account for the participant’s habitual PB engagement, rather than a one size fits all approach.

## Supporting information

S1 Table Effect of PB on fasting plasma LOG cholesterol concentration according to isotemporal substitution of one hour per day of SB or PA. (DOCX)

S2 Table Effect of PB on fasting plasma LOG triglyceride concentration according to isotemporal substitution of one hour per day of SB or PA. (DOCX)

S3 Table Effect of PB on fasting serum LOG LPL concentration according to isotemporal substitution of one hour per day of SB or PA. (DOCX)

S4 Table Effect of PB on fasting plasma glucose concentration according to isotemporal substitution of one hour per day of SB or PA. (DOCX)

S5 Table Effect of PB on fasting plasma HbA1c according to isotemporal substitution of one hour per day of SB or PA. (DOCX)

S6 Table Effect of PB on fasting serum LOG IL-6 concentration according to isotemporal substitution of one hour per day of SB or PA. (DOCX)

S7 Table Effect of PB on fasting serum LOG PIIINP concentration according to isotemporal substitution of one hour per day of SB or PA. (DOCX)

## References

[1] National Health Service. Physical activity guidelines for adults. 2013 [cited 2014 16th October 2014]. Available from: http://www.nhs.uk/Livewell/fitness/Pages/physical-activity-guidelines-for-adults.aspx.

[2] Nocon M, Hiemann T, Müller-Riemenschneider F, Thalau F, Roll S, Willich SN. Association of physical activity with all-cause and cardiovascular mortality: a systematic review and meta-analysis. European Journal of Cardiovascular Prevention & Rehabilitation. 2008;15(3):239–46. 18525377 10.1097/HJR.0b013e3282f55e09

[3] Townsend N, Bhatnagar P, Wilkins E, Wickramasinghe K, Rayner M. Cardiovascular disease statistics 2015. London: 2015.

[4] Ryan DJ, Wullems JA, Stebbings GK, Morse CI, Stewart CE, Onambele-Pearson GL. Reliability and validity of the international physical activity questionnaire compared to calibrated accelerometer cut-off points in the quantification of sedentary behaviour and physical activity in older adults. PloS one. 2018;13(4):e0195712 10.1371/journal.pone.0195712 29672534 PMC5908192

[5] Craig R, Mindell J, Hirani V. Health Survey for England 2008. Volume 1: Physical Activity and Fitness. Health Survey for England. 2009;1:8–395.

[6] Stamatakis E, Davis M, Stathi A, Hamer M. Associations between multiple indicators of objectively-measured and self-reported sedentary behaviour and cardiometabolic risk in older adults. Preventive medicine. 2012;54(1):82–7. 10.1016/j.ypmed.2011.10.009 22057055

[7] Pescatello LS, Murphy D, Costanzo D. Low-intensity physical activity benefits blood lipids and lipoproteins in older adults living at home. Age and Ageing. 2000;29(5):433–9. 10.1093/ageing/29.5.433 11108416

[8] Henson J, Yates T, Biddle SJ, Edwardson CL, Khunti K, Wilmot EG, et al Associations of objectively measured sedentary behaviour and physical activity with markers of cardiometabolic health. Diabetologia. 2013;56(5):1012–20. 10.1007/s00125-013-2845-9 23456209

[9] Mekary RA, Willett WC, Hu FB, Ding EL. Isotemporal substitution paradigm for physical activity epidemiology and weight change. American journal of epidemiology. 2009;170(4):519–27. 10.1093/aje/kwp163 19584129 PMC2733862

[10] Grgic J, Dumuid D, Bengoechea EG, Shrestha N, Bauman A, Olds T, et al Health outcomes associated with reallocations of time between sleep, sedentary behaviour, and physical activity: a systematic scoping review of isotemporal substitution studies. International Journal of Behavioral Nutrition and Physical Activity. 2018;15(1):69 10.1186/s12966-018-0691-3 30001713 PMC6043964

[11] Hamer M, Stamatakis E, Steptoe A. Effects of substituting sedentary time with physical activity on metabolic risk. Med Sci Sports Exerc. 2014;46(10):1946–50. 10.1249/MSS.0000000000000317 24674977 PMC4186723

[12] Healy GN, Winkler EA, Owen N, Anuradha S, Dunstan DW. Replacing sitting time with standing or stepping: associations with cardio-metabolic risk biomarkers. European heart journal. 2015:ehv308. 10.1093/eurheartj/ehv308 26228867

[13] Healy GN, Winkler EA, Brakenridge CL, Reeves MM, Eakin EG. Accelerometer-derived sedentary and physical activity time in overweight/obese adults with type 2 diabetes: cross-sectional associations with cardiometabolic biomarkers. PloS one. 2015;10(3):e0119140 10.1371/journal.pone.0119140 25775249 PMC4361561

[14] Ekblom-Bak E, Ekblom Ö, Bergström G, Börjesson M. Isotemporal substitution of sedentary time by physical activity of different intensities and bout lengths, and its associations with metabolic risk. European journal of preventive cardiology. 2016;23(9):967–74. 10.1177/2047487315619734 26635358

[15] Whitaker KM, Buman MP, Odegaard AO, Carpenter KC, Jacobs DR Jr, Sidney S, et al Sedentary Behaviors and Cardiometabolic Risk: An Isotemporal Substitution Analysis. American journal of epidemiology. 2017;187(2):181–9. 10.1093/aje/kwx209 PMC5860012 28595346

[16] Rosique-Esteban N, Díaz-López A, Martínez-González MA, Corella D, Goday A, Martínez JA, et al Leisure-time physical activity, sedentary behaviors, sleep, and cardiometabolic risk factors at baseline in the PREDIMED-PLUS intervention trial: A cross-sectional analysis. PloS one. 2017;12(3):e0172253 10.1371/journal.pone.0172253 28273154 PMC5342184

[17] Yates T, Henson J, Edwardson C, Dunstan D, Bodicoat DH, Khunti K, et al Objectively measured sedentary time and associations with insulin sensitivity: Importance of reallocating sedentary time to physical activity. Preventive medicine. 2015;76:79–83. 10.1016/j.ypmed.2015.04.005 25900801

[18] Buman M, Kurka J, Winkler E, Gardiner P, Hekler E, Healy G, et al Estimated replacement effects of accelerometer-derived physical activity and self-reported sleep duration on chronic disease biomarkers. Journal of Science and Medicine in Sport. 2012;15:S76.

[19] Ekblom-Bak E, Ekblom Ö, Bolam KA, Ekblom B, Bergström G, Börjesson M. SCAPIS pilot study: sitness, fitness and fatness—is sedentary time substitution by physical activity equally important for everyone’s markers of glucose regulation? Journal of Physical Activity and Health. 2016;13(7):697–703. 10.1123/jpah.2015-0611 26900674

[20] Dumuid D, Lewis L, Olds T, Maher C, Bondarenko C, Norton L. Relationships between older adults’ use of time and cardio-respiratory fitness, obesity and cardio-metabolic risk: a compositional isotemporal substitution analysis. Maturitas. 2018;110:104–10. 10.1016/j.maturitas.2018.02.003 29563028

[21] Ryan DJ W, J A, Stebbings G K, Morse C I, Stewart C E, Onambele-Pearson G L. Segregating the distinct effects of sedentary behaviour and physical activity on older adults’ cardiovascular structure and function: Part 2- Isotemporal substitution analysis Journal of physical activity & health. 2018:[In Press]. 10.1123/jpah.2017-0326 29580146

[22] Henson J, Davies MJ, Bodicoat DH, Edwardson CL, Gill JM, Stensel DJ, et al Breaking up prolonged sitting with standing or walking attenuates the postprandial metabolic response in post-menopausal women: a randomised acute study. 2016. 10.2337/dc15-1240 26628415

[23] Duvivier BM, Schaper NC, Bremers MA, van Crombrugge G, Menheere PP, Kars M, et al Minimal intensity physical activity (standing and walking) of longer duration improves insulin action and plasma lipids more than shorter periods of moderate to vigorous exercise (cycling) in sedentary subjects when energy expenditure is comparable. PloS one. 2013;8(2):e55542 10.1371/journal.pone.0055542 23418444 PMC3572053

[24] Fenton T. Regional gross disposable household income (GDHI): 1997 to 2015. In: Statistics OfN, editor. England: ONS; 2017.

[25] Office for National Statistics. 2011 census analysis, local area analysis of qualifications across england and wales. In: ONS, editor. England 2014.

[26] Ryan DJ, Wullems J A, Stebbings G K, Morse C I, Stewart C E, Onambele-Pearson G L. Segregating the distinct effects of sedentary behaviour and physical activity on older adults’ cardiovascular structure and function: Part 1- Linear regression analysis approach. Journal of physical activity & health. 2018;17(7):499–509. 10.1123/jpah.2017-0325 29485928

[27] Wullems JA, Verschueren SM, Degens H, Morse CI, Onambélé GL. Performance of thigh-mounted triaxial accelerometer algorithms in objective quantification of sedentary behaviour and physical activity in older adults. PloS one. 2017;12(11):e0188215 10.1371/journal.pone.0188215 29155839 PMC5695782

[28] Coqueiro RdS, Santos MC, Neto JdSL, Queiroz BMd, Brügger NAJ, Barbosa AR. Validity of a portable glucose, total cholesterol, and triglycerides multi-analyzer in adults. Biological research for nursing. 2014;16(3):288–94. 10.1177/1099800413495953 23871994

[29] Phillips CG, Nwagbo Y, Ashton K. Analytical evaluation of POCT HbA1c instruments—The 3rd EFLM-UEMS Congress. Clin Chem Lab Med. 2014;52(11):eA205—aE379.

[30] Mekary RA, Lucas M, Pan A, Okereke OI, Willett WC, Hu FB, et al Isotemporal substitution analysis for physical activity, television watching, and risk of depression. American journal of epidemiology. 2013;178(3):474–83. 10.1093/aje/kws590 23785112 PMC3727339

[31] Furberg CD, Adams HP, Applegate WB, Byington RP, Espeland MA, Hartwell T, et al Effect of lovastatin on early carotid atherosclerosis and cardiovascular events. Asymptomatic Carotid Artery Progression Study (ACAPS) Research Group. Circulation. 1994;90(4):1679–87. 10.1161/01.cir.90.4.1679 7734010

[32] Bakris GL, Fonseca V, Katholi RE, McGill JB, Messerli FH, Phillips RA, et al Metabolic effects of carvedilol vs metoprolol in patients with type 2 diabetes mellitus and hypertension: a randomized controlled trial. Jama. 2004;292(18):2227–36. 10.1001/jama.292.18.2227 15536109

[33] McIntyre RS, Soczynska JK, Konarski JZ, Kennedy SH. The effect of antidepressants on lipid homeostasis: a cardiac safety concern? Expert opinion on drug safety. 2006;5(4):523–37. 10.1517/14740338.5.4.523 16774491

[34] Tsuboi I, Tanaka H, Nakao M, Shichijo S, Itoh K. Nonsteroidal anti-inflammatory drugs differentially regulate cytokine production in human lymphocytes: Up-regulation of TNF, IFN-γ and IL-2, in contrast to down-regulation of IL-6 production. Cytokine. 1995;7(4):372–9. 10.1006/cyto.1995.0047 8589268

[35] Verschuren WM, Jacobs DR, Bloemberg BP, Kromhout D, Menotti A, Aravanis C, et al Serum total cholesterol and long-term coronary heart disease mortality in different cultures: Twenty-five—year follow-up of the seven countries study. Jama. 1995;274(2):131–6. 7596000

[36] Hokanson JE, Austin MA. Plasma triglyceride level is a risk factor for cardiovascular disease independent of high-density lipoprotein cholesterol level: a metaanalysis of population-based prospective studies. Journal of cardiovascular risk. 1996;3(2):213–9. 8836866

[37] Motoyama M, Sunami Y, Kinoshita F, Irie T, Sasaki J, Arakawa K, et al The effects of long-term low intensity aerobic training and detraining on serum lipid and lipoprotein concentrations in elderly men and women. European journal of applied physiology and occupational physiology. 1995;70(2):126–31. 10.1007/bf00361539 7768234

[38] Petibois C, Cassaigne A, Gin H, Déléris Gr. Lipid profile disorders induced by long-term cessation of physical activity in previously highly endurance-trained subjects. The Journal of Clinical Endocrinology & Metabolism. 2004;89(7):3377–84. 15240618 10.1210/jc.2003-031311

[39] Mora-Rodriguez R, Ortega J, Hamouti N, Fernandez-Elias V, Garcia-Prieto JC, Guadalupe-Grau A, et al Time-course effects of aerobic interval training and detraining in patients with metabolic syndrome. Nutrition, Metabolism and Cardiovascular Diseases. 2014;24(7):792–8. 10.1016/j.numecd.2014.01.011 24656853

[40] Shirai K, Itoh Y, Sasaki H, Totsuka M, Murano T, Watanabe H, et al The effect of insulin sensitizer, troglitazone, on lipoprotein lipase mass in preheparin serum. Diabetes research and clinical practice. 1999;46(1):35–41. 10.1016/s0168-8227(99)00063-7 10580614

[41] Watanabe H, Miyashita Y, Murano T, Hiroh Y, Itoh Y, Shirai K. Preheparin serum lipoprotein lipase mass level: the effects of age, gender, and types of hyperlipidemias. Atherosclerosis. 1999;145(1):45–50. 10.1016/s0021-9150(99)00012-x 10428294

[42] Hitsumoto T, Ohsawa H, Uchi T, Noike H, Kanai M, Yoshinuma M, et al Preheparin serum lipoprotein lipase mass is negatively related to coronary atherosclerosis. Atherosclerosis. 2000;153(2):391–6. 10.1016/s0021-9150(00)00413-5 11164428

[43] Rip J, Nierman MC, Wareham NJ, Luben R, Bingham SA, Day NE, et al Serum Lipoprotein Lipase Concentration and Risk for Future Coronary Artery Disease. Arteriosclerosis, thrombosis, and vascular biology. 2006;26(3):637–42. 10.1161/01.ATV.0000201038.47949.56 16373616

[44] Kantor MA, Cullinane EM, Sady SP, Herbert PN, Thompson PD. Exercise acutely increases high density lipoprotein-cholesterol and lipoprotein lipase activity in trained and untrained men. Metabolism. 1987;36(2):188–92. 10.1016/0026-0495(87)90016-3 3807790

[45] Hamilton MT, Etienne J, McClure WC, Pavey BS, Holloway AK. Role of local contractile activity and muscle fiber type on LPL regulation during exercise. American Journal of Physiology-Endocrinology And Metabolism. 1998;275(6):E1016–E22. 10.1152/ajpendo.1998.275.6.E1016 9843744

[46] Nikkilä EA, Taskinen M-R, Rehunen S, Härkönen M. Lipoprotein lipase activity in adipose tissue and skeletal muscle of runners: relation to serum lipoproteins. Metabolism. 1978;27(11):1661–71. 10.1016/0026-0495(78)90288-3 212665

[47] Goldberg IJ. Lipoprotein lipase and lipolysis: central roles in lipoprotein metabolism and atherogenesis. Journal of lipid research. 1996;37(4):693–707. 8732771

[48] Saito K, Sakurabayashi I, Manabe M. Serum lipoprotein lipase in healthy subjects: effects of gender and age, and relationships to lipid parameters. Annals of clinical biochemistry. 1998;35(6):733–8. 9838986 10.1177/000456329803500605

[49] Zilversmit DB. Atherogenesis: a postprandial phenomenon. Circulation. 1979;60(3):473–85. 10.1161/01.cir.60.3.473 222498

[50] Sarwar N, Gao P, Seshasai S, Gobin R, Kaptoge S, Di Angelantonio E, et al Emerging Risk Factors Collaboration Diabetes mellitus, fasting blood glucose concentration, and risk of vascular disease: a collaborative meta-analysis of 102 prospective studies. Lancet. 2010;375(9733):2215–22. 10.1016/S0140-6736(10)60484-9 20609967 PMC2904878

[51] Bailey DP, Locke CD. Breaking up prolonged sitting with light-intensity walking improves postprandial glycemia, but breaking up sitting with standing does not. Journal of Science and Medicine in Sport. 2014;18(3):294–8. 10.1016/j.jsams.2014.03.008 24704421

[52] Pulsford RM, Blackwell J, Hillsdon M, Kos K. Intermittent walking, but not standing, improves postprandial insulin and glucose relative to sustained sitting: a randomised cross-over study in inactive middle-aged men. Journal of Science and Medicine in Sport. 2016. 10.1016/j.jsams.2016.08.012 27633397

