# Supplementary material for: Using isotemporal substitution to predict the effects of changing physical behaviour on older adults’ cardio-metabolic profiles
Source: PLoS One. 2019 Oct 23;14(10):e0224223. doi: 10.1371/journal.pone.0224223 (PMC6808553; doi:10.1371/journal.pone.0224223)
Supplement: S1 Table — (DOCX) [file pone.0224223.s001.docx]

|  | SB | | | Standing | | | LIPA | | | sMVPA | | | _10_MVPA | | | Total PB | | |  |  |  |  |  |  |
| --- | --- | --- | --- | --- | --- | --- | --- | --- | --- | --- | --- | --- | --- | --- | --- | --- | --- | --- | --- | --- | --- | --- | --- | --- |
| **Replaced PB** | b | 95% CI | | b | 95% CI | | b | 95% CI | | b | 95% CI | | b | 95% CI | | b | 95% CI | |  |  | |  |  | |
| SB - Model 1 | Replaced | | | **-0.13** | **-0.25** | **-0.01** | **0.09** | **0.00** | **0.17** | -0.03 | -0.09 | 0.04 | -0.06 | -0.32 | 0.19 | 0.00 | -0.06 | 0.05 |  |  |  |  |  |  |
| SB - Model 2 |  |  |  | **-0.13** | **-0.23** | **-0.02** | 0.06 | -0.02 | 0.14 | -0.03 | -0.09 | 0.02 | -0.11 | -0.34 | 0.11 | 0.00 | -0.05 | 0.05 |  |  |  |  |  |  |
| Standing - Model 1 | 0.08 | -0.03 | 0.20 | Replaced | | | 0.13 | -0.03 | 0.29 | 0.06 | -0.08 | 0.19 | 0.03 | -0.25 | 0.31 | -0.07 | -0.17 | 0.04 |  |  |  |  |  |  |
| Standing - Model 2 | 0.09 | -0.02 | 0.19 |  |  |  | 0.11 | -0.04 | 0.25 | 0.05 | -0.07 | 0.18 | -0.02 | -0.28 | 0.23 | -0.07 | -0.17 | 0.02 |  |  |  |  |  |  |
| LIPA - Model 1 | -0.09 | -0.17 | 0.00 | **-0.22** | **-0.40** | **-0.04** | Replaced | | | -0.12 | -0.24 | 0.01 | -0.15 | -0.41 | 0.11 | 0.08 | -0.01 | 0.18 |  |  |  |  |  |  |
| LIPA - Model 2 | -0.06 | -0.14 | 0.02 | **-0.19** | **-0.35** | **-0.02** |  |  |  | -0.09 | -0.21 | 0.02 | -0.17 | -0.41 | 0.06 | 0.06 | -0.03 | 0.14 |  |  |  |  |  |  |
| sMVPA - Model 1 | -0.01 | -0.06 | 0.05 | **-0.15** | **-0.28** | **-0.01** | 0.05 | -0.06 | 0.16 | Replaced | | | -0.14 | -0.40 | 0.12 | 0.04 | -0.02 | 0.09 |  |  |  |  |  |  |
| sMVPA - Model 2 | 0.00 | -0.05 | 0.05 | **-0.14** | **-0.27** | **-0.02** | 0.03 | -0.07 | 0.13 |  |  |  | -0.18 | -0.42 | 0.06 | 0.03 | -0.02 | 0.08 |  |  |  |  |  |  |
| _10_MVPA - Model 1 | 0.06 | -0.19 | 0.31 | -0.08 | -0.36 | 0.20 | 0.17 | -0.09 | 0.42 | 0.04 | -0.24 | 0.31 | Replaced | | | -0.06 | -0.32 | 0.19 |  |  |  |  |  |  |
| _10_MVPA - Model 2 | 0.13 | -0.09 | 0.35 | -0.01 | -0.26 | 0.24 | 0.19 | -0.03 | 0.42 | 0.10 | -0.14 | 0.34 |  |  |  | -0.13 | -0.36 | 0.09 |  |  |  |  |  |  |

**S1 Table** Effect of PB on fasting plasma LOG cholesterol concentration according to isotemporal substitution of one hour per day of SB or PA.

Model 1 No covariates included. Model 2 Covariates included – Directly CVD medication, (in)directly CVD medication.

Model 1 No covariates included. Model 2 Covariates included – Direct CVD medication, (in)directly CVD medication.

**Bold** indicates significant changes in cardio-metabolic parameter, *p* ≤ 0.05.
