# Supplementary material for: Using isotemporal substitution to predict the effects of changing physical behaviour on older adults’ cardio-metabolic profiles
Source: PLoS One. 2019 Oct 23;14(10):e0224223. doi: 10.1371/journal.pone.0224223 (PMC6808553; doi:10.1371/journal.pone.0224223)
Supplement: S2 Table — (DOCX) [file pone.0224223.s002.docx]

**S2 Table** Effect of PB on fasting plasma LOG triglyceride concentration according to isotemporal substitution of one hour per day of SB or PA.

|  | SB | | | Standing | | | LIPA | | | sMVPA | | | _10_MVPA | | | Total PB | | |
| --- | --- | --- | --- | --- | --- | --- | --- | --- | --- | --- | --- | --- | --- | --- | --- | --- | --- | --- |
| **Replaced PB** | b | 95% CI | | b | 95% CI | | b | 95% CI | | b | 95% CI | | b | 95% CI | | b | 95% CI | |
| SB - Model 1 | Replaced | | | -0.04 | -0.26 | 0.18 | -0.04 | -0.20 | 0.12 | -0.12 | -0.24 | 0.00 | **-0.68** | **-1.15** | **-0.21** | 0.05 | -0.05 | 0.15 |
| SB - Model 2 |  |  |  |  |  |  |  |  |  |  |  |  |  |  |  |  |  |  |
| Standing - Model 1 | 0.00 | -0.21 | 0.21 | Replaced | | | -0.08 | -0.37 | 0.22 | -0.12 | -0.36 | 0.13 | **-0.67** | **-1.18** | **-0.16** | 0.07 | -0.12 | 0.26 |
| Standing - Model 2 |  |  |  |  |  |  |  |  |  |  |  |  |  |  |  |  |  |  |
| LIPA - Model 1 | 0.04 | -0.12 | 0.20 | 0.00 | -0.33 | 0.34 | Replaced | | | -0.08 | -0.31 | 0.16 | **-0.64** | **-1.12** | **-0.16** | 0.01 | -0.16 | 0.18 |
| LIPA - Model 2 |  |  |  |  |  |  |  |  |  |  |  |  |  |  |  |  |  |  |
| sMVPA - Model 1 | 0.08 | -0.03 | 0.19 | 0.02 | -0.23 | 0.27 | 0.02 | -0.19 | 0.22 | Replaced | | | **-0.71** | **-1.19** | **-0.23** | 0.01 | -0.09 | 0.12 |
| sMVPA - Model 2 |  |  |  |  |  |  |  |  |  |  |  |  |  |  |  |  |  |  |
| _10_MVPA - Model 1 | **0.62** | **0.16** | **1.07** | **0.58** | **0.08** | **1.08** | **0.58** | **0.12** | **1.05** | 0.49 | 0.00 | 0.99 | Replaced | | | **-0.56** | **-1.03** | **-0.10** |
| _10_MVPA - Model 2 |  |  |  |  |  |  |  |  |  |  |  |  |  |  |  |  |  |  |

Model 1 No covariates included. Model 2 Covariates included – NA.

**Bold** indicates significant changes in cardio-metabolic parameter, *p* ≤ 0.05.
