# Supplementary material for: Using isotemporal substitution to predict the effects of changing physical behaviour on older adults’ cardio-metabolic profiles
Source: PLoS One. 2019 Oct 23;14(10):e0224223. doi: 10.1371/journal.pone.0224223 (PMC6808553; doi:10.1371/journal.pone.0224223)
Supplement: S3 Table — (DOCX) [file pone.0224223.s003.docx]

**S3 Table** Effect of PB on fasting serum LOG LPL concentration according to isotemporal substitution of one hour per day of SB or PA.

|  | SB | | | Standing | | | LIPA | | | sMVPA | | | _10_MVPA | | | Total PB | | |  |  |  |
| --- | --- | --- | --- | --- | --- | --- | --- | --- | --- | --- | --- | --- | --- | --- | --- | --- | --- | --- | --- | --- | --- |
| **Replaced PB** | b | 95% CI | | b | 95% CI | | b | 95% CI | | b | 95% CI | | b | 95% CI | | b | 95% CI | |  |  | |
| SB - Model 1 | Replaced | | | -0.02 | -0.73 | 0.68 | -0.26 | -0.76 | 0.25 | -0.07 | -0.46 | 0.31 | 0.08 | -1.39 | 1.55 | **0.33** | **0.01** | **0.64** |  |  |  |
| SB - Model 2 |  |  |  | 0.11 | -0.56 | 0.78 | -0.42 | -0.93 | 0.09 | -0.11 | -0.48 | 0.25 | -0.17 | -1.57 | 1.23 | **0.41** | **0.11** | **0.72** |  |  |  |
| Standing - Model 1 | 0.19 | -0.45 | 0.83 | Replaced | | | 0.08 | -0.84 | 0.99 | 0.11 | -0.67 | 0.89 | 0.20 | -1.38 | 1.79 | 0.07 | -0.54 | 0.68 |  |  |  |
| Standing - Model 2 | 0.13 | -0.48 | 0.73 |  |  |  | -0.08 | -0.96 | 0.80 | 0.02 | -0.72 | 0.77 | -0.10 | -1.63 | 1.42 | 0.18 | -0.40 | 0.77 |  |  |  |
| LIPA - Model 1 | 0.26 | -0.25 | 0.76 | 0.23 | -0.83 | 1.30 | Replaced | | | 0.18 | -0.54 | 0.90 | 0.34 | -1.19 | 1.87 | 0.07 | -0.47 | 0.61 |  |  |  |
| LIPA - Model 2 | 0.42 | -0.09 | 0.93 | 0.54 | -0.50 | 1.57 |  |  |  | 0.31 | -0.38 | 1.00 | 0.26 | -1.19 | 1.70 | -0.01 | -0.54 | 0.52 |  |  |  |
| sMVPA - Model 1 | 0.28 | -0.08 | 0.63 | 0.27 | -0.56 | 1.09 | 0.27 | -0.38 | 0.92 | Replaced | | | 0.72 | -0.83 | 2.28 | -0.16 | -0.48 | 0.17 |  |  |  |
| sMVPA - Model 2 | 0.21 | -0.14 | 0.55 | 0.38 | -0.39 | 1.16 | -0.16 | -0.82 | 0.51 |  |  |  | 0.33 | -1.12 | 1.77 | 0.09 | -0.26 | 0.44 |  |  |  |
| _10_MVPA - Model 1 | -0.35 | -1.76 | 1.05 | -0.31 | -1.84 | 1.22 | -0.58 | -2.04 | 0.87 | -0.45 | -1.98 | 1.08 | Replaced | | | 0.68 | -0.76 | 2.12 |  |  |  |
| _10_MVPA - Model 2 | -0.17 | -1.52 | 1.18 | 0.00 | -1.48 | 1.48 | -0.54 | -1.92 | 0.85 | -0.30 | -1.76 | 1.16 |  |  |  | 0.57 | -0.80 | 1.94 |  |  |  |

Model 1 No covariates included. Model 2 Covariates included – Inflammatory + (in)directly CVD medication.

**Bold** indicates significant changes in cardio-metabolic parameter, *p* ≤ 0.05.
