# Supplementary material for: Using isotemporal substitution to predict the effects of changing physical behaviour on older adults’ cardio-metabolic profiles
Source: PLoS One. 2019 Oct 23;14(10):e0224223. doi: 10.1371/journal.pone.0224223 (PMC6808553; doi:10.1371/journal.pone.0224223)
Supplement: S4 Table — (DOCX) [file pone.0224223.s004.docx]

**S4 Table** Effect of PB on fasting plasma glucose concentration according to isotemporal substitution of one hour per day of SB or PA.

|  | SB | | | Standing | | | LIPA | | | sMVPA | | | _10_MVPA | | | Total PB | | |
| --- | --- | --- | --- | --- | --- | --- | --- | --- | --- | --- | --- | --- | --- | --- | --- | --- | --- | --- |
| **Replaced PB** | b | 95% CI | | b | 95% CI | | b | 95% CI | | b | 95% CI | | b | 95% CI | | b | 95% CI | |
| SB - Model 1 | Replaced | | | -0.02 | -0.84 | 0.80 | -0.31 | -0.90 | 0.29 | -0.07 | -0.52 | 0.38 | -0.47 | -2.21 | 1.26 | 0.10 | -0.28 | 0.48 |
| SB - Model 2 |  |  |  |  |  |  |  |  |  |  |  |  |  |  |  |  |  |  |
| Standing - Model 1 | 0.20 | -0.56 | 0.96 | Replaced | | | 0.07 | -1.02 | 1.15 | 0.12 | -0.79 | 1.04 | -0.31 | -2.20 | 1.58 | -0.18 | -0.89 | 0.53 |
| Standing - Model 2 |  |  |  |  |  |  |  |  |  |  |  |  |  |  |  |  |  |  |
| LIPA - Model 1 | 0.31 | -0.29 | 0.90 | 0.29 | -0.95 | 1.53 | Replaced | | | 0.24 | -0.62 | 1.10 | -0.16 | -1.94 | 1.61 | -0.21 | -0.85 | 0.43 |
| LIPA - Model 2 |  |  |  |  |  |  |  |  |  |  |  |  |  |  |  |  |  |  |
| sMVPA - Model 1 | 0.07 | -0.33 | 0.47 | 0.02 | -0.89 | 0.92 | -0.20 | -0.94 | 0.54 | Replaced | | | -0.46 | -2.20 | 1.27 | 0.04 | -0.33 | 0.41 |
| sMVPA - Model 2 |  |  |  |  |  |  |  |  |  |  |  |  |  |  |  |  |  |  |
| _10_MVPA - Model 1 | 0.38 | -1.27 | 2.04 | 0.42 | -1.41 | 2.24 | 0.06 | -1.63 | 1.75 | 0.28 | -1.52 | 2.09 | Replaced | | | -0.28 | -1.96 | 1.41 |
| _10_MVPA - Model 2 |  |  |  |  |  |  |  |  |  |  |  |  |  |  |  |  |  |  |

Model 1 No covariates included. Model 2 Covariates included - NA.
