# Supplementary material for: Using isotemporal substitution to predict the effects of changing physical behaviour on older adults’ cardio-metabolic profiles
Source: PLoS One. 2019 Oct 23;14(10):e0224223. doi: 10.1371/journal.pone.0224223 (PMC6808553; doi:10.1371/journal.pone.0224223)
Supplement: S5 Table — (DOCX) [file pone.0224223.s005.docx]

**S5 Table** Effect of PB on fasting plasma HbA1c according to isotemporal substitution of one hour per day of SB or PA.

|  | SB | | | Standing | | | LIPA | | | sMVPA | | | _10_MVPA | | | Total PB | | |
| --- | --- | --- | --- | --- | --- | --- | --- | --- | --- | --- | --- | --- | --- | --- | --- | --- | --- | --- |
| **Replaced PB** | b | 95% CI | | b | 95% CI | | b | 95% CI | | b | 95% CI | | b | 95% CI | | b | 95% CI | |
| SB - Model 1 | Replaced | | | 0.18 | -0.13 | 0.50 | -0.27 | -0.55 | 0.00 | 0.08 | -0.14 | 0.29 | -0.23 | -1.16 | 0.69 | -0.05 | -0.20 | 0.10 |
| SB - Model 2 |  |  |  |  |  |  |  |  |  |  |  |  |  |  |  |  |  |  |
| Standing - Model 1 | -0.18 | -0.50 | 0.13 | Replaced | | | -0.46 | -0.99 | 0.08 | -0.11 | -0.50 | 0.29 | -0.42 | -1.37 | 0.53 | 0.13 | -0.21 | 0.47 |
| Standing - Model 2 |  |  |  |  |  |  |  |  |  |  |  |  |  |  |  |  |  |  |
| LIPA - Model 1 | 0.27 | 0.00 | 0.55 | 0.46 | -0.08 | 0.99 | Replaced | | | 0.35 | -0.03 | 0.73 | 0.04 | -0.92 | 1.00 | -0.33 | -0.60 | -0.05 |
| LIPA - Model 2 |  |  |  |  |  |  |  |  |  |  |  |  |  |  |  |  |  |  |
| sMVPA - Model 1 | -0.08 | -0.29 | 0.14 | 0.11 | -0.29 | 0.50 | -0.35 | -0.73 | 0.03 | Replaced | | | -0.31 | -1.33 | 0.71 | 0.02 | -0.23 | 0.28 |
| sMVPA - Model 2 |  |  |  |  |  |  |  |  |  |  |  |  |  |  |  |  |  |  |
| _10_MVPA - Model 1 | 0.18 | -0.73 | 1.09 | 0.35 | -0.59 | 1.28 | -0.10 | -1.04 | 0.84 | 0.25 | -0.76 | 1.27 | Replaced | | | -0.23 | -1.14 | 0.69 |
| _10_MVPA - Model 2 |  |  |  |  |  |  |  |  |  |  |  |  |  |  |  |  |  |  |

Model 1 No covariates included. Model 2 Covariates included – NA
