# Supplementary material for: Using isotemporal substitution to predict the effects of changing physical behaviour on older adults’ cardio-metabolic profiles
Source: PLoS One. 2019 Oct 23;14(10):e0224223. doi: 10.1371/journal.pone.0224223 (PMC6808553; doi:10.1371/journal.pone.0224223)
Supplement: S6 Table — (DOCX) [file pone.0224223.s006.docx]

**S6 Table** Effect of PB on fasting serum LOG IL-6 concentration according to isotemporal substitution of one hour per day of SB or PA.

|  | SB | | | Standing | | | LIPA | | | sMVPA | | | _10_MVPA | | | Total PB | | |
| --- | --- | --- | --- | --- | --- | --- | --- | --- | --- | --- | --- | --- | --- | --- | --- | --- | --- | --- |
| **Replaced PB** | b | 95% CI | | b | 95% CI | | b | 95% CI | | b | 95% CI | | b | 95% CI | | b | 95% CI | |
| SB - Model 1 | Replaced | | | 0.02 | -0.55 | 0.59 | 0.18 | -0.24 | 0.60 | -0.23 | -0.54 | 0.07 | 0.58 | -0.67 | 1.82 | -0.02 | -0.28 | 0.24 |
| SB - Model 2 |  |  |  |  |  |  |  |  |  |  |  |  |  |  |  |  |  |  |
| Standing - Model 1 | -0.02 | -0.53 | 0.50 | Replaced | | | 0.17 | -0.57 | 0.91 | -0.25 | -0.87 | 0.37 | 0.56 | -0.75 | 1.87 | -0.01 | -0.49 | 0.48 |
| Standing - Model 2 |  |  |  |  |  |  |  |  |  |  |  |  |  |  |  |  |  |  |
| LIPA - Model 1 | -0.18 | -0.60 | 0.24 | -0.16 | -1.03 | 0.71 | Replaced | | | -0.42 | -1.00 | 0.17 | 0.40 | -0.91 | 1.70 | 0.16 | -0.28 | 0.60 |
| LIPA - Model 2 |  |  |  |  |  |  |  |  |  |  |  |  |  |  |  |  |  |  |
| sMVPA - Model 1 | 0.15 | -0.12 | 0.43 | 0.19 | -0.45 | 0.82 | 0.22 | -0.30 | 0.73 | Replaced | | | 0.62 | -0.65 | 1.89 | -0.09 | -0.34 | 0.17 |
| sMVPA - Model 2 |  |  |  |  |  |  |  |  |  |  |  |  |  |  |  |  |  |  |
| _10_MVPA - Model 1 | -0.52 | -1.73 | 0.69 | -0.46 | -1.76 | 0.83 | -0.27 | -1.53 | 0.99 | -0.77 | -2.08 | 0.53 | Replaced | | | 0.49 | -0.75 | 1.74 |
| _10_MVPA - Model 2 |  |  |  |  |  |  |  |  |  |  |  |  |  |  |  |  |  |  |

Model 1 No covariates included. Model 2 Covariates included - NA
