# Supplementary material for: Using isotemporal substitution to predict the effects of changing physical behaviour on older adults’ cardio-metabolic profiles
Source: PLoS One. 2019 Oct 23;14(10):e0224223. doi: 10.1371/journal.pone.0224223 (PMC6808553; doi:10.1371/journal.pone.0224223)
Supplement: S7 Table — (DOCX) [file pone.0224223.s007.docx]

**S7 Table** Effect of PB on fasting serum LOG PIIINP concentration according to isotemporal substitution of one hour per day of SB or PA.

|  | SB | | | Standing | | | LIPA | | | sMVPA | | | _10_MVPA | | | Total PB | | |
| --- | --- | --- | --- | --- | --- | --- | --- | --- | --- | --- | --- | --- | --- | --- | --- | --- | --- | --- |
| **Replaced PB** | b | 95% CI | | b | 95% CI | | b | 95% CI | | b | 95% CI | | b | 95% CI | | b | 95% CI | |
| SB - Model 1 | Replaced | | | -0.02 | -0.90 | 0.87 | 0.15 | -0.50 | 0.79 | 0.04 | -0.42 | 0.50 | 0.01 | -1.80 | 1.83 | 0.27 | -0.12 | 0.65 |
| SB - Model 2 |  |  |  |  |  |  |  |  |  |  |  |  |  |  |  |  |  |  |
| Standing - Model 1 | 0.14 | -0.65 | 0.93 | Replaced | | | 0.39 | -0.73 | 1.52 | 0.18 | -0.79 | 1.15 | 0.11 | -1.84 | 2.05 | 0.08 | -0.65 | 0.82 |
| Standing - Model 2 |  |  |  |  |  |  |  |  |  |  |  |  |  |  |  |  |  |  |
| LIPA - Model 1 | -0.15 | -0.79 | 0.50 | -0.16 | -1.50 | 1.18 | Replaced | | | -0.10 | -1.00 | 0.79 | -0.13 | -2.03 | 1.77 | 0.41 | -0.29 | 1.12 |
| LIPA - Model 2 |  |  |  |  |  |  |  |  |  |  |  |  |  |  |  |  |  |  |
| sMVPA - Model 1 | 0.08 | -0.32 | 0.48 | 0.15 | -0.82 | 1.13 | 0.30 | -0.48 | 1.08 | Replaced | | | 0.42 | -1.41 | 2.24 | 0.05 | -0.31 | 0.42 |
| sMVPA - Model 2 |  |  |  |  |  |  |  |  |  |  |  |  |  |  |  |  |  |  |
| _10_MVPA - Model 1 | 0.07 | -1.63 | 1.78 | 0.03 | -1.84 | 1.90 | 0.28 | -1.49 | 2.05 | 0.10 | -1.77 | 1.96 | Replaced | | | 0.21 | -1.54 | 1.95 |
| _10_MVPA - Model 2 |  |  |  |  |  |  |  |  |  |  |  |  |  |  |  |  |  |  |

Model 1 No covariates included. Model 2 Covariates included - NA.
